# Managing Dysregulated Vitamin D Metabolism in CKD: Time to Update Conventional Wisdom?

**DOI:** 10.34067/KID.0000000982

**Published:** 2025-08-26

**Authors:** Charles W. Bishop, Akhtar Ashfaq, John Choe, Keith C. Norris, Stuart M. Sprague

**Affiliations:** 1 Renal Division, OPKO Health, Inc., Miami, Florida; 2 David Geffen School of Medicine, UCLA, Los Angeles, California; 3 NorthShore University Health System-University of Chicago, Pritzker School of Medicine, Evanston, Illinois

**Keywords:** CKD, clinical trial, dialysis, metabolism, nephrology, parathyroid hormone, renal progression, vitamin D, CKD-MBD

Circulating 25-hydroxyvitamin D (25D) and 1,25-dihydroxyvitamin D (1,25D, the active vitamin D hormone) decline as CKD advances, becoming insufficient in the absence of repletion and driving secondary hyperparathyroidism. The current clinical practice guideline does not specify a target for parathyroid hormone (PTH) due to the absence of supporting randomized clinical trial data. However, it suggests correction of vitamin D insufficiency using treatment strategies recommended for the general population^1^ targeting 25D levels of 20–30 ng/ml and using dietary vitamin D supplements (cholecalciferol or ergocalciferol). They focus on dose, not exposure, overlooking physiologic barriers making it difficult to raise serum 25D to targeted levels, such as adipose related increases in volume of distribution, impaired hepatic activation in CKD and obesity, and increased catabolism due to upregulation of CYP24A1 (cytochrome P450 24-hydroxylase), the vitamin D catabolic enzyme.^2^

Supplements have become standard-of-care treatment for dysregulated vitamin D metabolism without clear evidence that they sufficiently raise serum 25D levels to control elevated PTH.^1^ Real world data show that 25D levels are inadequate in most patients with CKD, despite customary treatment.^2^ Ineffective supplementation allows PTH to rise, prompting a switch to hormone treatment (*e.g*., oral calcitriol) and squandering the opportunity for early and more effective 25D repletion.

Vitamin D hormone therapy increases a patient's “calcium burden” and associated risk of hypercalcemia.^1,3^ Accordingly, the current guideline^1^ suggests avoiding its routine use in nondialysis patients. Oral administration delivers hormone directly to vitamin D receptors (VDR) localized in the upper small intestine, increasing active absorption of dietary calcium.^3^ Hormone therapy also increases serum phosphorus and fibroblast growth factor 23 (FGF23), which have been linked to faster CKD progression and induction of CYP24A1 expression in target tissues, decreasing therapeutic responsiveness.^3^

Conventional wisdom encompasses long-held tenets that kidneys are the only significant source of 1,25D and that loss of renal CYP27B1 (cytochrome P450 25D-1*α*-hydroxylase) with declining kidney function eventually necessitates hormone replacement therapy.

CYP27B1 is broadly expressed beyond the kidneys, including in the parathyroid glands. When renal CYP27B1 declines in advancing CKD, its extrarenal counterparts can be engaged therapeutically to become major alternative sources of 1,25D, thereby restoring normal vitamin D metabolism, as illustrated by trials with extended-release calcifediol (ERC) in adults with stage 3, 4 or 5 CKD.^4,5^

Extrarenal CYP27B1 receives 25D substrate largely by passive diffusion which requires a high concentration gradient across cell membranes.^4^ Intracrine production of 1,25D increases with rising serum 25D concentrations and is not limited by feedback mechanisms regulating renal CYP27B1.^4,6^ Data from two randomized controlled trials (RCTs) with ERC in patients with stage 3 or 4 CKD suggest that raising serum 25D to ≥50 ng/ml is required to engage extrarenal CYP27B1, as the frequency of 30% reductions in elevated PTH became higher as 25D rose above that level.^5^ PTH-lowering response rates were agnostic to CKD stage indicating that comparable delivery of 1,25D to intracellular VDR in parathyroids was achieved despite large differences in kidney function.

A recent randomized clinical trial in chronic hemodialysis patients found that raising serum 25D concentrations to ≥50 ng/ml with ERC normalized circulating 1,25D from undetectable or low pretreatment levels.^4^ Further 25D elevation increased serum 1,25D in near-linear fashion to levels reaching or exceeding the upper end of the reference range, providing additional evidence of an absence of feedback inhibition of extrarenal 1,25D production. Trials in surgically anephric patients have also indicated that serum 1,25D can derive from extrarenal CYP27B1 in proportion to available 25D substrate.^4^ These observations impugn the widely held belief that serum 25D repletion for dysregulated vitamin D metabolism becomes futile as CKD advances**.**

Conventional wisdom cautions against raising serum 25D to ≥50 ng/ml, thereby hindering therapeutic engagement of extrarenal CYP27B1. In 2011, the Institute of Medicine expressed safety concerns about such 25D exposures, including increased risk of cancer, toxicity, and all-cause mortality.^7^ A carcinogenicity study required for regulatory approval of ERC found no neoplastic changes attributable to daily subcutaneous administration of calcifediol at ≤33 *µ*g/kg per day for 26 weeks to rasH2 transgenic mice (data on file). Meaningfully increased rates of hypercalcemia, hypercalciuria, elevated serum phosphorus or FGF23, or treatment-emergent adverse events were not observed with ERC versus placebo in RCTs, wherein serum 25D concentrations averaged nearly 70 ng/mL.^8^ Longer RCTs are needed to assess rates of all-cause mortality with sustained 25D exposures of ≥50 ng/ml.

Others have postulated higher toxicity thresholds for serum 25D, ranging from 60 to 250 ng/ml, all of which remain unsubstantiated.^4^ Clinical evidence indicates that these toxicity estimates are low for ERC as toxicity depends more on the daily rate of serum 25D elevation during repletion than on the absolute exposure achieved.^4,8^ Toxicity likely derives from 1,25D exposure, not 25D exposure, given the far greater affinity (500-fold to 1000-fold) of active hormone for the VDR. Unfortunately, safety concerns have discouraged effective management of dysregulated vitamin D metabolism in CKD with appropriate 25D replacement therapy.

Most patients with CKD are overweight, and serum 25D exposures of ≥50 ng/ml can be especially difficult to achieve with cholecalciferol or ergocalciferol in overweight patients.^2^ These fat-soluble dietary supplements accumulate preferentially in adipose tissue. They have low affinities for the serum vitamin D binding protein and are poorly mobilized from adipose tissue into circulation for hepatic activation.^2^ Hepatic 25-hydroxylase (*e.g*., CYP27A1) activity is reduced in both CKD and obesity, blunting the intended elevation of serum 25D with supplements.^2^

**Figure 1:**
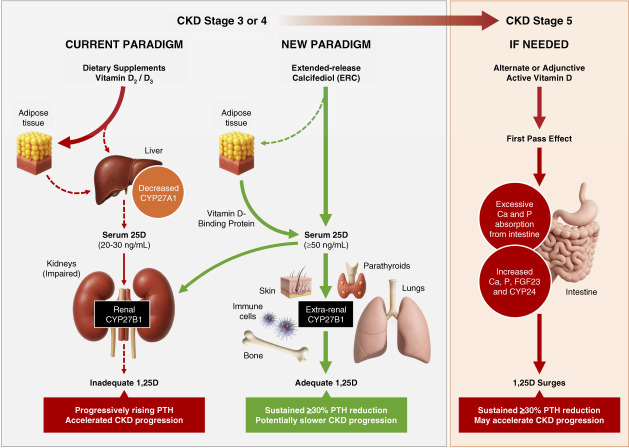
**Proposed update to current treatment paradigm for dysregulated vitamin D metabolism in CKD.** Extrarenal expression of CYP27B1 occurs in skin, parathyroids, lungs, bone, and immune cells (as depicted) and in many other tissues. 1,25D, 1,25-dihydroxyvitamin D; 25D, 25-hydroxyvitamin D; Ca, calcium; CYP24, cytochrome P450 24-hydroxylase; CYP27A1, cytochrome P450 25-hydroxylase; CYP27B1, cytochrome P450 25D-1*α*-hydroxylase; FGF23, fibroblast growth factor 23; P, phosphorus; PTH, parathyroid hormone.

Calcifediol, in contrast, readily achieves 25D exposures of ≥50 ng/ml because it does not require hepatic activation, is relatively water soluble, and avidly binds to D binding protein, reducing accumulation in adipose tissue.^2^ Gradual delivery of calcifediol with ERC avoids significant CYP24A1 upregulation and catabolic loss of substrate.^9^ Furthermore, ERC slowly releases inactive prohormone (calcifediol) and is absorbed with greatly reduced first pass activation compared with hormone therapies,^3^ avoiding clinically relevant increases in serum calcium, phosphorus, and FGF23 compared with placebo.^8^ Recently published data indicate that a sustained 30% reduction of elevated PTH with ERC was associated with a reduced rate of CKD progression,^10^ whereas hormone replacement therapy accelerated progression.^3^

Data suggest that it is time to consider changes to the current treatment paradigm for managing dysregulated vitamin D metabolism in CKD, as delineated in Figure 1. First, the commonly used target for serum 25D of 20–30 ng/ml should be increased to ≥50 ng/ml so that sufficient 1,25D can be produced by extrarenal CYP27B1. Second, initial treatment should consider ERC rather than cholecalciferol or ergocalciferol to reliably and sufficiently raise serum 25D, given the high prevalence of adiposity. Third, the daily dose of ERC should target a sustained ≥30% reduction of elevated PTH to increase the possibility of slowing CKD progression. Restoration of normal vitamin D metabolism can be effectively accomplished in many patients with CKD by slowly raising serum 25D with ERC, generating sufficient extrarenal 1*α*-hydroxylation and subsequently normalizing serum 1,25D levels.

## Supplementary Material
