# Supplementary material for: Managing Dysregulated Vitamin D Metabolism in CKD: Time to Update Conventional Wisdom?
Source: Kidney360. 2025 Aug 26;6(11):2000–2. doi: 10.34067/KID.0000000982 (PMC12626653; doi:10.34067/KID.0000000982)
Supplement: Supplementary file 1 [file kidney360-6-2000-s001.pdf]

ASN American Society of Nephrology
JASN Journal of the American Society of Nephrology
KIDNEY360 Accessing Our World From Every Angle
CJASN Clinical Journal of the American Society of Nephrology

ASN Journal Disclosure Form

As per ASN journal policy, I have disclosed any financial relationships or commitments I have held in the past 36 months as included below. I have listed my Current Employer below to indicate there is a relationship requiring disclosure. If no relationship exists, my Current Employer is not listed.

A. Ashfaq reports the following:
Employer: opko pharmacueticals; Ownership Interest: opko pharmacueticals; Research Funding: OPKO Pharmaceuticals; Patents or Royalties: Receive Royalty fro UpToDate for "TINU Syndrome".; and Other Interests or Relationships: full time employee of opko pharmacueticals.

I understand that the information above will be published within the journal article, if accepted, and that failure to comply and/or to accurately and completely report the potential financial conflicts of interest could lead to the following: 1) Prior to publication, article rejection, or 2) Post-publication, sanctions ranging from, but not limited to, issuing a correction, reporting the inaccurate information to the authors' institution, banning authors from submitting work to ASN journals for varying lengths of time, and/or retraction of the published work.

Name: Akhtar Ashfaq
Manuscript ID: K360-2025-000691R2
Manuscript Title: Managing deregulated vitamin d metabolism in CKD: time to update the conventional wisdom?
Date of Completion: August 20, 2025
Disclosure Updated Date: August 20, 2025

ASN American Society of Nephrology | JASN Journal of the American Society of Nephrology | KIDNEY360 Accessing Our World From Every Angle | CJASN Clinical Journal of the American Society of Nephrology

ASN Journal Disclosure Form

As per ASN journal policy, I have disclosed any financial relationships or commitments I have held in the past 36 months as included below. I have listed my Current Employer below to indicate there is a relationship requiring disclosure. If no relationship exists, my Current Employer is not listed.

C. Bishop reports the following:
Employer: Opko Health; Ownership Interest: Opko Health; Research Funding: OPKO Health; Japan Tobacco International; Patents or Royalties: OPKO Health; Advisory or Leadership Role: OPKO Health; and Speakers Bureau: OPKO Heath.

I understand that the information above will be published within the journal article, if accepted, and that failure to comply and/or to accurately and completely report the potential financial conflicts of interest could lead to the following: 1) Prior to publication, article rejection, or 2) Post-publication, sanctions ranging from, but not limited to, issuing a correction, reporting the inaccurate information to the authors' institution, banning authors from submitting work to ASN journals for varying lengths of time, and/or retraction of the published work.

Name: Charles W. Bishop
Manuscript ID: K360-2025-000691R2
Manuscript Title: Managing dysregulated vitamin D metabolism in CKD: Time to update conventional wisdom?
Date of Completion: August 19, 2025
Disclosure Updated Date: August 19, 2025

ASN American Society of Nephrology | JASN Journal of the American Society of Nephrology | KIDNEY360 Accessing Our World From Every Angle | CJASN Clinical Journal of the American Society of Nephrology

ASN Journal Disclosure Form

As per ASN journal policy, I have disclosed any financial relationships or commitments I have held in the past 36 months as included below. I have listed my Current Employer below to indicate there is a relationship requiring disclosure. If no relationship exists, my Current Employer is not listed.

J. Choe reports the following:
Employer: OPKO Health Inc.; and Ownership Interest: OPKO Health Inc.

I understand that the information above will be published within the journal article, if accepted, and that failure to comply and/or to accurately and completely report the potential financial conflicts of interest could lead to the following: 1) Prior to publication, article rejection, or 2) Post-publication, sanctions ranging from, but not limited to, issuing a correction, reporting the inaccurate information to the authors' institution, banning authors from submitting work to ASN journals for varying lengths of time, and/or retraction of the published work.

Name: John Choe
Manuscript ID: K360-2025-000691R1
Manuscript Title: Managing dysregulated vitamin D metabolism in CKD: Time to update conventional wisdom?
Date of Completion: July 24, 2025
Disclosure Updated Date: July 24, 2025

ASN
American Society of Nephrology
JASN
Journal of the American Society of Nephrology
KIDNEY360
Accessing Our World From Every Angle
CJASN
Clinical Journal of the American Society of Nephrology

ASN Journal Disclosure Form

As per ASN journal policy, I have disclosed any financial relationships or commitments I have held in the past 36 months as included below. I have listed my Current Employer below to indicate there is a relationship requiring disclosure. If no relationship exists, my Current Employer is not listed.

K. Norris reports the following:
Consultancy: Atlantis Healthcare - Compliance, research and quality care for dialysis and CKD care in Puerto Rico; Phizer - Advisory board on increasing diversity in clinical trials; Patents or Royalties: Up-To-Date chapter on the use of race and ethnicity in medicine.; Advisory or Leadership Role: Ethnicity and Disease; JASN; CJASN; AAMC; Atlantis Healthcare; AAKP; NKF-KEEP; ISN; ESRD Network Forum, NIDDK Council, NIH Council of Councils; and Other Interests or Relationships: AAKP; NKF; ESRD Network Forum; ASN; SGIM.

I understand that the information above will be published within the journal article, if accepted, and that failure to comply and/or to accurately and completely report the potential financial conflicts of interest could lead to the following: 1) Prior to publication, article rejection, or 2) Post-publication, sanctions ranging from, but not limited to, issuing a correction, reporting the inaccurate information to the authors' institution, banning authors from submitting work to ASN journals for varying lengths of time, and/or retraction of the published work.

Name: Keith C. Norris
Manuscript ID: K360-2025-000691R1
Manuscript Title: Managing dysregulated vitamin D metabolism in CKD: Time to update conventional wisdom
Date of Completion: July 24, 2025
Disclosure Updated Date: July 24, 2025

ASN American Society of Nephrology | JASN Journal of the American Society of Nephrology | KIDNEY360 Accessing Our World From Every Angle | CJASN Clinical Journal of the American Society of Nephrology

ASN Journal Disclosure Form

As per ASN journal policy, I have disclosed any financial relationships or commitments I have held in the past 36 months as included below. I have listed my Current Employer below to indicate there is a relationship requiring disclosure. If no relationship exists, my Current Employer is not listed.

S. Sprague reports the following:
Employer: Endeavor Health-NorthShore University HealthSystem, Univ of Chicago Pritzker School of Medicine; Consultancy: OPKO, Vifor, Amgen, Fresenius, Litholink Corp, Shire, Horizon, Ardelyx, Bayer; Ownership Interest: Individually owned stocks; Amgen, Apple, IBM, Walgreens, First Australia Fund, Coca Cola, Baxter, Bristol Myers; Research Funding: Amgen, Ardelyx, Reata, OPKO, Takeda, Amylot; Honoraria: Vifor, Fresenius, Amgen, OPKO, Ardelyx, Bayer; Advisory or Leadership Role: National Kidney Foundation of Illinois, American Journal of Nephrology, International Federation of Clinical Chemistry and Laboratory Medicine-Work Group for Parathyroid Hormone, American Association of Endocrine Surgeons; and Speakers Bureau: Amgen; OPKO; Fresenius, Bayer.

I understand that the information above will be published within the journal article, if accepted, and that failure to comply and/or to accurately and completely report the potential financial conflicts of interest could lead to the following: 1) Prior to publication, article rejection, or 2) Post-publication, sanctions ranging from, but not limited to, issuing a correction, reporting the inaccurate information to the authors' institution, banning authors from submitting work to ASN journals for varying lengths of time, and/or retraction of the published work.

Name: Stuart M. Sprague
Manuscript ID: K360-2025-000691R2
Manuscript Title: Managing dysregulated vitamin D metabolism in CKD: Time to update conventional wisdom?
Date of Completion: August 20, 2025
Disclosure Updated Date: August 20, 2025
